# VALID regimen of voriconazole optimizes therapeutic target attainment and minimizes adverse events in patients with liver dysfunction

**DOI:** 10.3389/fphar.2026.1895947

**Published:** 2026-08-12

**Authors:** Jiakai Li, Yichang Zhao, Huaiyuan Liu, Yukun Zhang, Jing Ma, Yongfang Jiang, Jingjing Zhao, Bikui Zhang, Miao Yan

**Affiliations:** 1 Department of Pharmacy, the Second Xiangya Hospital of Central South University, Changsha, China; 2 International Research Center for Precision Medicine, Transformative Technology and Software Services, Hunan, China; 3 School of Basic Medicine and Clinical Pharmacy, China Pharmaceutical University, Nanjing, China; 4 Department of Infectious Disease, the Second Xiangya Hospital of Central South University, Changsha, China; 5 Inner Mongolia Medical University, Hohho, China

**Keywords:** dosing regimen, liver dysfunction, therapeutic drug monitoring, total bilirubin, voriconazole

## Abstract

**Objective:**

Liver dysfunction affects drug metabolism, including voriconazole, used for treating fungal infections. This study evaluated the effectiveness and safety of a voriconazole dosing regimen adjusted for liver dysfunction (VALID) compared with routine clinician-directed dosing.

**Methods:**

This observational, non-interventional study included hospitalized patients with clinically diagnosed liver dysfunction, mainly including liver cirrhosis and acute-on-chronic liver failure, who received voriconazole for the prevention or treatment of invasive fungal infection between January 2020 and December 2023. Patients were assigned to the VALID or control group according to their actual initial dosing regimen. Propensity score matching was performed using 1:1 nearest-neighbor matching without replacement and a caliper value of 0.05. Voriconazole trough concentrations were measured using liquid chromatography. CYP2C19 phenotype, when available, and liver function-related indicators were assessed as covariates influencing voriconazole exposure.

**Results:**

A total of 182 patients with liver dysfunction and 375 voriconazole trough concentration measurements were initially included, including 39 patients in the VALID group and 143 patients in the control group. After propensity score matching, 39 patients from each group were included for between-group comparisons. The VALID group had a significantly higher initial target trough concentration attainment rate than the control group (89.7% vs. 48.7%, P < 0.001) and a lower initial trough concentration [2.46 (1.85–3.04) mg/L vs. 4.13 (2.78–6.48) mg/L, P = 0.0003]. The VALID group also had a lower incidence of voriconazole‐related adverse events (15.4% vs. 38.5%, P = 0.022), while clinical efficacy and dose adjustment did not differ significantly between the two groups. CYP2C19 phenotype, liver function‐related indicators, coagulation‐related indicators, and daily dose were associated with voriconazole trough concentrations.

**Conclusion:**

The TBIL-based VALID regimen showed potential to improve target trough concentration attainment and reduce voriconazole-related adverse events in patients with liver dysfunction while maintaining comparable efficacy. However, this regimen should be interpreted as a preliminary initial dosing strategy and still requires validation in larger multicenter prospective studies. TDM and individualized dose adjustment remain necessary during treatment.

## 1 Introduction

The global burden of liver disease remains a significant health challenge, encompassing conditions such as autoimmune hepatitis (AIH), hepatitis B and C, and metabolic dysfunction-associated steatotic liver disease (MASLD) (Wang et al., 2024; Patil et al., 2024; Ou et al., 2024; Danpanichkul et al., 2024). Furthermore, patients with liver dysfunction are highly susceptible to fungal infections due to compromised immune systems, leading to higher infection rates and severe outcomes (Gupta et al., 2024; Ferrarese et al., 2020). Notably, invasive fungal infections significantly affect survival and post-transplant outcomes, with a prevalence of about 10.2% in cirrhosis patients and higher rates in transplant recipients, exacerbating the disease burden and increasing mortality risk (Miloh et al., 2017; Verma et al., 2022; van Delden et al., 2020; Corzo-León et al., 2018). Therefore, effective management including early identification and treatment of fungal infections, enhanced monitoring, and preventive measures like antifungal prophylaxis is needed (Hassan et al., 2018; Wadi and Denning, 2018).

Voriconazole is a crucial antifungal medication strongly recommended by various guidelines and studies for the treatment of invasive fungal infections (Takesue et al., 2022; Maertens et al., 2018; Gómez-López, 2020; Ferreras-Antolín et al., 2019; Garcia-Vidal et al., 2019; Husain and Camargo, 2019; Wang et al., 2020). It is a second-generation triazole antifungal agent widely used for treating invasive fungal infections, including invasive aspergillosis and *Candida* species. It is available in both intravenous and oral formulations, with well-absorbed oral bioavailability. Voriconazole’s pharmacokinetics are complex, involving polymorphic cytochrome P450 enzymes (CYP2C19, CYP2C9, and CYP3A4), resulting in significant interpatient and intrapatient variability (Malani et al., 2015; Schulz et al., 2019). Due to its narrow therapeutic range and unpredictable serum levels, therapeutic drug monitoring (TDM) is essential to ensure efficacy and minimize toxicity. Although different guidelines have proposed slightly different target ranges, the target trough concentration range used in the present study was defined as 0.5–5.0 mg/L according to the Chinese guideline for individualized voriconazole therapy (Takesue et al., 2022; Chen et al., 2018). Besides, precise dosage adjustments are essential in patients with hepatic impairment to optimize therapeutic efficacy while minimizing potential adverse effects. Current research underscores the importance of thorough assessment of physiological and biochemical parameters to guide voriconazole dosing. Key strategies for dosage adjustment include considerations based on total bilirubin (TBIL) levels and the Child-Pugh score. Elevated TBIL levels, which indicate liver function status, necessitate a reduction in voriconazole dosage to prevent excessive drug concentrations and potential toxicity (Tang et al., 2019; Tang et al., 2021). The Child-Pugh score, which incorporates variables such as bilirubin, albumin levels, and coagulation profiles, is used to evaluate hepatic function. Patients with a high score (Class C) indicate severe hepatic impairment, requiring significant dosage reductions (Tang et al., 2021; Wang et al., 2021). Further, the patient’s body weight and end-stage liver disease scores, such as the MELD score, significantly influence voriconazole pharmacokinetics. Patients characterized by lower body weight or higher MELD scores typically require reduced dosages (Wu et al., 2024). Regular monitoring of plasma drug concentrations is pivotal in adjusting dosages appropriately to ensure both safety and effectiveness of the treatment (Wang et al., 2022; Zhao et al., 2021a). Genetic variability, especially in the CYP2C19 enzyme, also affects the metabolism of voriconazole. Patients with variants leading to slow metabolism may necessitate adjustments in dosing (Lin et al., 2018; Li et al., 2017). Additionally, drug interactions, particularly when co-administered with proton pump inhibitors and other common medications, may require further adjustments in voriconazole dosing (Zhao et al., 2021b; Yan et al., 2018). Although existing research offers sophisticated models for dosage adjustments, the TBIL-based dosing optimization strategy proposed by Tang et al., derived from Monte Carlo simulations, still mandates further clinical validation, which can ensure the efficacy and safety of these strategies across diverse clinical scenarios, ensuring the applicability of dosage recommendations and enhancing overall treatment success rates while reducing adverse events (Tang et al., 2021; Wang et al., 2021). Therefore, this study was designed to clinically validate the TBIL-based voriconazole dosing strategy, namely, the VALID regimen, as an initial dosing strategy for patients with liver dysfunction. The primary objective was to evaluate its target attainment, efficacy, safety, and need for subsequent dose adjustment in clinical practice.

## 2 Methods

### 2.1 Patients, inclusion and exclusion criteria

This study was approved by the Ethics Committee of the Second Xiangya Hospital of Central South University [approval number: 2022 Ethical Review CR No. (066)]. This was an observational, non-interventional study with retrospective data collection and prospective clinical observation. The study did not involve any additional intervention beyond routine clinical care, and voriconazole dosing was determined by the treating clinicians. Written informed consent was obtained from the patients or their legal representatives according to the ethics approval requirements. All patient data were de-identified before analysis. From January 2020 to December 2023, patients with liver dysfunction hospitalized in the Second Xiangya Hospital of Central South University were eligible if they met the following criteria: (1) aged 16 years or older; (2) clinically diagnosed with liver dysfunction before or at the initiation of voriconazole therapy, defined as a documented diagnosis of liver cirrhosis, acute-on-chronic liver failure, or clinically diagnosed hepatic dysfunction accompanied by abnormal liver function-related indicators, such as elevated bilirubin or transaminases, decreased albumin, or coagulation abnormalities; (3) treated with voriconazole for the prevention or treatment of IFI for a minimum of 3 days; (4) at least one voriconazole trough concentration measurement taken during hospitalization. The exclusion criteria included: (1) pregnancy or lactation; (2) patients with voriconazole allergy or intolerance; (3) combined CYP450 inducers or inhibitors such as rifampicin, isoniazid, and phenytoin during voriconazole use; (4) poor medication compliance; (5) other conditions not suitable for inclusion.

### 2.2 Dosing regimen

The voriconazole dosing regimen for patients with liver dysfunction was determined by the treating clinicians. Patients were divided into the VALID group and the control group according to their actual initial dosing regimen. In the VALID group, the initial dosing regimen was determined according to the baseline TBIL level assessed before or at the initiation of voriconazole therapy. Subsequent dose adjustment was performed according to TDM results, changes in liver function, adverse events, and clinical judgment when necessary. The specific dosing regimens were as follows.

#### 2.2.1 The VALID group (TBIL-based dosing regimen)

For the VALID group, the voriconazole dosing was adjusted according to the levels of total bilirubin (TBIL) to optimize treatment for patients with varying degrees of liver dysfunction. This subgroup approach was designed to assess the practicality and efficacy of a tailored pharmacotherapy regimen based on specific liver function parameters. The dosing strategy was structured as follows:

TBIL -based dosing regimen for Voriconazole (VALID Regimen).

**Table:** 

Total bilirubin (μmol/L)	Loading dose	Maintenance dose
TBIL≤51	400 mg q12 h	100 mg q12 h
51<TBIL≤171	200 mg q12 h	100 mg qd/50 mg q12 h
TBIL>171	200 mg q12 h	50 mg qd

q12 h: every 12 h; qd: every day.

This TBIL-based dosing regimen was derived from previous population pharmacokinetic and Monte Carlo simulation studies in patients with liver dysfunction (Tang et al., 2021; Wang et al., 2021).

#### 2.2.2 Control group

According to the voriconazole prescribing information, for patients with mild to moderate liver dysfunction, including Child-Pugh class A and B cirrhosis, the loading dose was unchanged at 400 mg q12 h, and the maintenance dose was reduced by half to 100 mg q12 h. Because no specific recommended dose is provided in the prescribing information for patients with Child-Pugh class C liver dysfunction, the initial dosing regimen in these patients was determined by the treating clinicians based on comprehensive clinical judgment, including liver disease severity, infection severity, body weight, concomitant medications, and expected safety risk. The actual initial voriconazole dosing regimens, including loading dose, maintenance dose, initial daily maintenance dose, and formulation, were extracted and summarized in the propensity score-matched cohort.

### 2.3 Data collection

A complete statistical table of patient information was designed, and the following information was collected from the hospital information system after the patients were enrolled: (1) demographic information: gender, age, body weight, etc. (2) medication information: voriconazole formulation, dose, dosing interval, dose adjustment, and adverse events, etc. (3) liver function, renal function, blood routine test, etc.

### 2.4 Propensity score matching (PSM)

Propensity score matching was performed to reduce baseline imbalance between the VALID group and the control group. The nearest-neighbor matching method was used, with a caliper value of 0.05. A 1:1 matching strategy without replacement was performed using the sklearn package in the Python Anaconda Navigator environment. Baseline characteristics before and after matching were compared between the two groups to evaluate the improvement in baseline comparability. Between-group comparisons of target attainment, efficacy, safety, and dose adjustment were performed in the propensity score-matched cohort.

### 2.5 Measurement of voriconazole trough concentration

Venous blood was collected for TDM 3–7 days after the first dose, 30 min before the next dose, or 3–7 days after dose adjustment. Voriconazole trough concentrations were measured using an automatic two-dimensional liquid chromatograph (Demeter Instrument Co. Ltd., Changsha, China). The absolute recovery was 88.16%–93.58%, the relative recovery was 94.17%–105.26%, and the intra- and inter-day precision values were 1.94%–2.22% and 2.15%–6.78%, respectively. The chromatographic conditions were as follows: an ASTON FRO C18 column (100 mm × 3.0 mm, 5 μm, ANAX) with a mobile phase of 20 mmol/L ammonium acetate-acetonitrile (48:52, v/v) at a flow rate of 1.0 mL/min, and an ASTON HD C18 column (150 mm × 4.6 mm, 5 μm, ANAX) with a mobile phase of 40 mmol/L ammonium acetate-acetonitrile (85:15, v/v) at a flow rate of 1.2 mL/min. The injection volume was 200 μL, the column temperature was 45 °C, the detection wavelength was 273 nm, and the linear detection range was 0.35–11.26 mg/L.

### 2.6 CYP2C19 genotyping and phenotype assignment

For patients with available blood samples, CYP2C19 genotyping was performed after voriconazole trough concentration measurement. Genomic DNA was extracted and purified from whole blood samples using the E. Z.N.A. SQ Blood DNA Kit II (Omega BioTek, Norcross, GA, USA). DNA concentration and purity were measured using a NanoDrop 2000 spectrophotometer. CYP2C19 genotypes were determined by Sanger sequencing using an ABI3730xl DNA Analyzer (Applied Biosystems, USA). CYP2C19 phenotypes were assigned according to genotype results and classified as normal metabolizers, intermediate metabolizers, or poor metabolizers.

### 2.7 Outcomes

#### 2.7.1 Primary outcome

The primary outcome was target attainment of voriconazole trough concentration in the VALID and control groups. According to the Chinese guideline for individualized voriconazole therapy, the target trough concentration range was defined as 0.5–5.0 mg/L (Chen et al., 2018). Because some international guidelines recommend slightly different target ranges, such as 1.0–5.5 mg/L, the potential influence of different target windows was considered in the interpretation of the results.

#### 2.7.2 Secondary outcomes

- Efficacy was assessed based on clinical manifestations, radiological findings, and microbiological results according to previous criteria and local clinical practice. For patients receiving voriconazole for the treatment of invasive fungal infection, treatment effectiveness was defined as complete or partial improvement in clinical manifestations, radiological findings, and microbiological results. Stable disease, disease progression, or infection-related death was classified as treatment failure. For prophylactic or empirical use, effectiveness was defined as no occurrence of invasive fungal infection, no discontinuation due to adverse events, and survival at the end of treatment. Because part of the outcome assessment relied on medical record review, efficacy assessment was not blinded to treatment group allocation.
- Safety was evaluated by systematically recording voriconazole-related adverse events and treatment modifications, including dose adjustment, frequency adjustment, and discontinuation. Adverse events included, but were not limited to, neurotoxicity, hepatotoxicity, visual disturbance, skin reactions, gastrointestinal reactions, renal toxicity, and hematological abnormalities.
- Dose adjustment was defined as any adjustment in voriconazole dose or dosing interval during therapy.

### 2.8 Statistical analysis

IBM SPSS Statistics 25.0 (IBM Corporation, NY, USA) and GraphPad Prism 9.0.0 (GraphPad Software, LLC, USA) were used to analyze and visualize the data, respectively. Quantitative data were compared using Student’s t-test or one-way ANOVA for normally distributed variables, and the Mann-Whitney U test or Kruskal–Wallis test for non-normally distributed variables, as appropriate. Categorical data were analyzed using the χ^2^ test or Fisher’s exact test. Multiple linear regression analysis was performed using the stepwise method. A two-sided P < 0.05 was considered statistically significant.

The initial target attainment analysis was conducted at the patient level using the first available trough concentration after treatment initiation. The overall target attainment analysis was conducted at the concentration-measurement level using all available trough concentration measurements during therapy. Between-group comparisons of target attainment, efficacy, safety, and dose adjustment were performed in the propensity score-matched cohort.

The analysis of factors influencing voriconazole trough concentration was conducted using the full cohort. To address the potential influence of repeated trough concentration measurements from the same patient, a patient-level sensitivity analysis was performed using only the first available trough concentration from each patient. For this sensitivity analysis, target attainment was reassessed in the propensity score-matched cohort, and the associations between initial voriconazole trough concentration and key covariates were re-evaluated in the full cohort at the patient level.

## 3 Results

### 3.1 Baseline characteristics

A total of 182 patients with liver dysfunction and 375 voriconazole trough concentration measurements were initially included in this study. Among them, 39 patients with 71 trough concentration measurements were assigned to the VALID group, and 143 patients with 304 trough concentration measurements were assigned to the control group. The demographic and baseline characteristics before matching are shown in Supplementary Table 1. Because significant baseline differences were observed between the two groups, especially in liver function-related indicators, propensity score matching was performed to improve baseline comparability. After 1:1 propensity score matching, no statistically significant differences were observed in the main baseline characteristics between the VALID and control groups (Table 1). Therefore, the matched cohort of 78 patients was used for comparisons of target attainment, efficacy, safety, and dose adjustment. For the analysis of factors influencing voriconazole trough concentrations, the full cohort of 182 patients and 375 trough concentration measurements was used. The actual initial voriconazole dosing regimens in the propensity score-matched cohort are summarized in Supplementary Table 3. As expected, the initial maintenance dose distribution differed between the two groups. The VALID group showed a more standardized and TBIL-stratified initial maintenance dose pattern, whereas the control group showed a higher and more heterogeneous initial maintenance dose distribution, reflecting routine clinician-directed dosing practice.

**TABLE 1:** Baseline characteristics of included patients after propensity score matching.

Parameters^1^	VALID group (n = 39)	Control group (n = 39)	*P*-value
Gender (Male, %)	27 (69.2%)	33 (84.6%)	0.1069
Child-Pugh class (A:B:C)	2:17:20	4:12:22	0.4660
MELD score	15.6 ± 7.8^*^ (17.0, 9.1∼20.9)	17.3 ± 7.3^*^ (18.4, 12.3∼21.2)	0.3661
Age (Year)	57.9 ± 11.7^*^ (56.0, 49.0∼69.0)	53.7 ± 15.0^*^ (56.0, 46.0∼63.0)	0.1763
Body weight (kg)	62.3 ± 13.1^*^ (64.0, 52.0∼71.0)	58.9 ± 11.0^*^ (60.0, 50.0∼66.5)	0.2249
ALT (U/L)	101.4 ± 295.4 (27.7, 17.6∼61.0)	70.7 ± 70.0 (39.1, 27.5∼86.7)	0.0778
AST (U/L)	121.1 ± 311.6 (55.7, 28.1∼97.7)	84.0 ± 66.2 (66.4, 36.4∼115.6)	0.2872
TP (g/L)	59.2 ± 8.4^*^ (57.5, 52.6∼65.0)	59.8 ± 8.9^*^ (59.7, 54.1∼66.0)	0.7420
ALB (g/L)	29.4 ± 4.5 (29.6, 24.9∼33.0)	29.6 ± 4.9^*^ (30.1, 26.0∼33.1)	0.9006
GLO (g/L)	29.7 ± 7.9^*^ (28.5, 24.8∼33.8)	30.2 ± 8.2^*^ (29.4, 23.5∼35.6)	0.8022
TBIL (μmol/L)	114.5 ± 144.0 (48.2, 22.5∼138.5)	157.7 ± 157.9 (126.1, 36.8∼237.6)	0.1109
DBIL (μmol/L)	78.4 ± 105.2 (25.5, 14.6∼97.1)	113.7 ± 122.6 (81.3, 23.2∼173.9)	0.1087
TBA (μmol/L)	110.3 ± 121.3 (74.6, 24.1∼172.9)	191.0 ± 222.4 (135.8, 19.1∼265.8)	0.1573
BUN (mmol/L)	9.26 ± 7.23 (7.11, 4.58∼9.49)	9.37 ± 7.49 (5.75, 4.29∼10.98)	0.6144
CREA (μmol/L)	90.0 ± 57.4 (65.8, 58.9∼101.5)	104.8 ± 83.6 (80.2, 56.7∼104.2)	0.7026
UA (μmol/L)	284.5 ± 197.5 (215.0, 153.8∼399.9)	258.6 ± 151.9 (204.0, 140.4∼367.3)	0.6909
PT (s)	20.6 ± 5.9 (19.6, 16.9∼22.3)	19.2 ± 4.7^*^ (18.1, 15.8∼21.9)	0.2352
INR	1.80 ± 0.70 (1.69, 1.37∼1.97)	1.63 ± 0.49 (1.47, 1.23∼1.95)	0.2292
PTA (%)	50.2 ± 16.8^*^ (46.0, 40.0∼63.0)	55.8 ± 22.1 (54.0, 37.8∼70.3)	0.3751
PT ratio	1.57 ± 0.45 (1.50, 1.28∼1.70)	1.46 ± 0.35^*^ (1.36, 1.18∼1.71)	0.2252
WBC (×10^9^/L)	5.82 ± 3.65 (4.69, 3.43∼7.29)	8.08 ± 6.47 (5.95, 3.78∼10.52)	0.2044
RBC (×10^12^/L)	2.86 ± 0.70^*^ (2.78, 2.41∼3.19)	2.88 ± 0.71^*^ (2.76, 2.37∼3.44)	0.9030
PLT (×10^9^/L)	93.7 ± 66.5 (70.0, 40.0∼130.0)	98.3 ± 76.7 (77.0, 58.0∼123.0)	0.4011
HGB (g/L)	92.2 ± 23.5^*^ (90.0, 74.0∼104.0)	92.1 ± 22.8^*^ (87.0, 75.0∼110.0)	0.9961
NEUT# (×10^9^/L)	4.40 ± 3.42 (3.20, 2.28∼6.04)	6.23 ± 5.45 (4.06, 2.27∼8.47)	0.1530
NEUT% (%)	71.2 ± 12.0^*^ (69.3, 64.6∼80.0)	72.5 ± 14.4 (75.0, 59.4∼86.0)	0.6350
LYM# (×10^9^/L)	0.91 ± 0.56 (0.85, 0.50∼1.14)	1.06 ± 1.15 (0.80, 0.58∼1.24)	0.6893
LYM% (%)	18.6 ± 10.1^*^ (17.7, 11.3∼24.1)	17.0 ± 11.5 (13.7, 7.9∼25.5)	0.3605
PCT (μg/L)	1.071 ± 1.532 (0.330, 0.158∼1.545)	1.003 ± 1.106 (0.659, 0.368∼1.473)	0.2543
CRP (mg/L)	41.27 ± 46.99 (20.78, 10.42∼62.24)	27.62 ± 24.30 (23.39, 8.87∼36.68)	0.6858

1 Mean ± SD (Median, IQR).

^*^ Shows that the variable is normal distribution.

### 3.2 Initial and overall target attainment of voriconazole trough concentration

The target range of voriconazole trough concentration was defined as 0.5–5.0 mg/L. The initial target attainment analysis was conducted at the patient level using the first available trough concentration after treatment initiation for each patient. The initial and overall target attainment results are summarized in Table 2. In the matched cohort, 35 of 39 patients in the VALID group achieved the initial target trough concentration, with an initial target attainment rate of 89.7%. Two patients had initial trough concentrations below 0.5 mg/L, and two patients had initial trough concentrations above 5.0 mg/L. In the control group, 19 of 39 patients achieved the initial target trough concentration, with an initial target attainment rate of 48.7%; two patients had initial trough concentrations below 0.5 mg/L, and 18 patients had initial trough concentrations above 5.0 mg/L. The initial target attainment rate was significantly higher in the VALID group than in the control group (P < 0.001).

**TABLE 2:** Initial and overall target attainment of voriconazole trough concentrations.

Target attainment status	VALID group	Control group	Total	*P*-value
Initial trough concentration				
Target	35	19	54	P < 0.001
Out of target	4	20	24	
Total	39	39	78	
Overall trough concentration				
Target	62	34	96	P < 0.001
Out of target	9	33	42	
Total	71	67	138	

**FIGURE 1:**
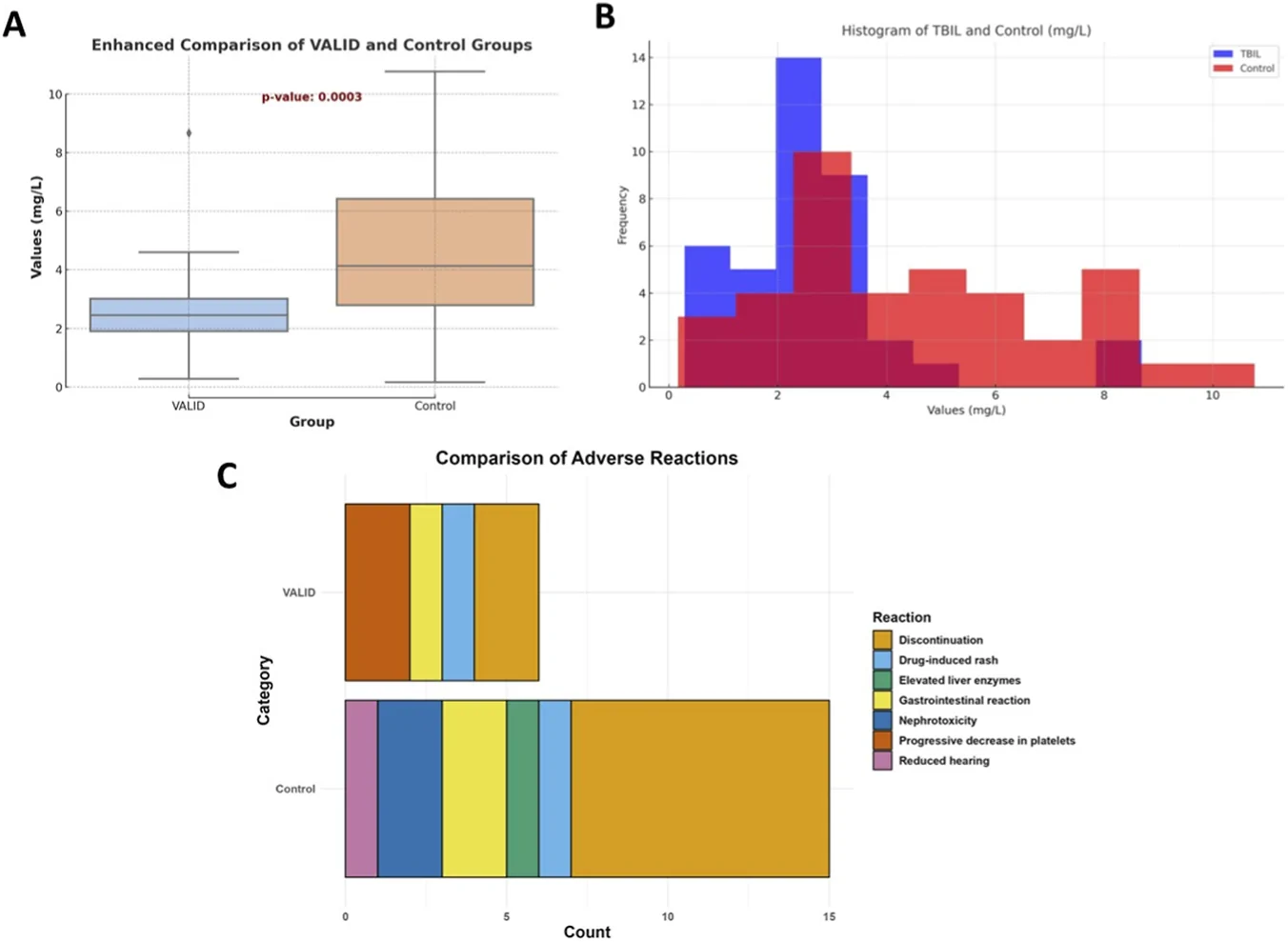
Comparison of voriconazole trough concentrations and adverse reactions between the VALID and control groups. **(A)** Boxplot comparing initial voriconazole trough concentrations between the VALID and control groups. The horizontal line within each box represents the median, and the whiskers indicate the dispersion of the data. The initial trough concentration was significantly lower in the VALID group than in the control group (P = 0.0003). **(B)** Overlaid histograms showing the frequency distributions of voriconazole trough concentrations in the VALID and control groups. The blue histogram represents the VALID group, and the red histogram represents the control group. **(C)** Stacked bar plot showing voriconazole‐related adverse events in the VALID and control groups. The total incidence of voriconazole‐related adverse events was 15.4% (6/39) in the VALID group and 38.5% (15/39) in the control group (P = 0.022).

The median initial voriconazole trough concentrations were 2.46 (1.85–3.04) mg/L in the VALID group and 4.13 (2.78–6.48) mg/L in the control group, respectively, with a significant difference between the two groups (Figure 1A, P = 0.0003).

The overall target attainment analysis was conducted at the concentration-measurement level using all available trough concentration measurements during therapy. In the matched cohort, the VALID group had 71 trough concentration measurements, while the control group had 67 trough concentration measurements. The overall target attainment rates were 87.3% (62/71) in the VALID group and 50.7% (34/67) in the control group, respectively (P < 0.001). The median trough concentrations across all measurements were 2.59 (1.79–3.36) mg/L in the VALID group and 4.13 (2.24–6.37) mg/L in the control group, respectively.

### 3.3 Efficacy of voriconazole

Based on the predefined efficacy criteria, 32 of 39 patients in the VALID group were classified as effective, and 7 were classified as ineffective, including 1 death. In the control group, 30 of 39 patients were classified as effective, 7 were classified as ineffective, and 2 were not evaluated because of the short duration of treatment. There was no significant difference in efficacy between the two groups (P = 0.913).

### 3.4 Safety of voriconazole

A total of 21 voriconazole related adverse events were observed in this study, including 6 cases in the VALID group and 15 cases in the control group. The most common adverse events were discontinuation of voriconazole due to excessive trough concentration (10 cases in total). Other adverse events included gastrointestinal reactions, rash, nephrotoxicity, and progressive platelet decline, as shown in Table 3. The incidence of voriconazole-related adverse events was significantly lower in the VALID group than in the control group [15.4% (6/39) vs. 38.5% (15/39), P = 0.022; Figure 1C].

**TABLE 3:** Voriconazole-related adverse events in the VALID and control groups.

Adverse event category	VALID group (number, %)	Control group (number, %)	P-value
Discontinued due to high VRZ trough concentration	2 (5.1)	8 (20.5)	​
Liver injury	0 (0.0)	1 (2.6)	​
Rash	1 (2.6)	1 (2.6)	​
Gastrointestinal reaction	1 (2.6)	2 (5.1)	​
Nephrotoxicity	0 (0.0)	2 (5.1)	​
Progressive decline of platelets	2 (5.1)	0 (0.0)	​
Decreased hearing	0 (0.0)	1 (2.6)	​
Total	6 (15.4)	15 (38.5)	P = 0.022

### 3.5 Dose adjustment of voriconazole

Of 39 patients in the VALID group, 10 patients underwent dose adjustment, of which 8 patients underwent one dose adjustment and 2 patients underwent two dose adjustments. Of the 39 patients in the control group, 17 underwent dose adjustments, including 13 patients who underwent 1 dose adjustment, 1 patient who underwent 2 dose adjustments, and 3 patients who underwent 3 dose adjustments. There was no statistically significant difference between the two groups in the proportion of patients requiring dose adjustment (P = 0.096) or in the number of dose adjustments (P = 0.089).

### 3.6 Factors influencing voriconazole trough concentrations

**FIGURE 2:**
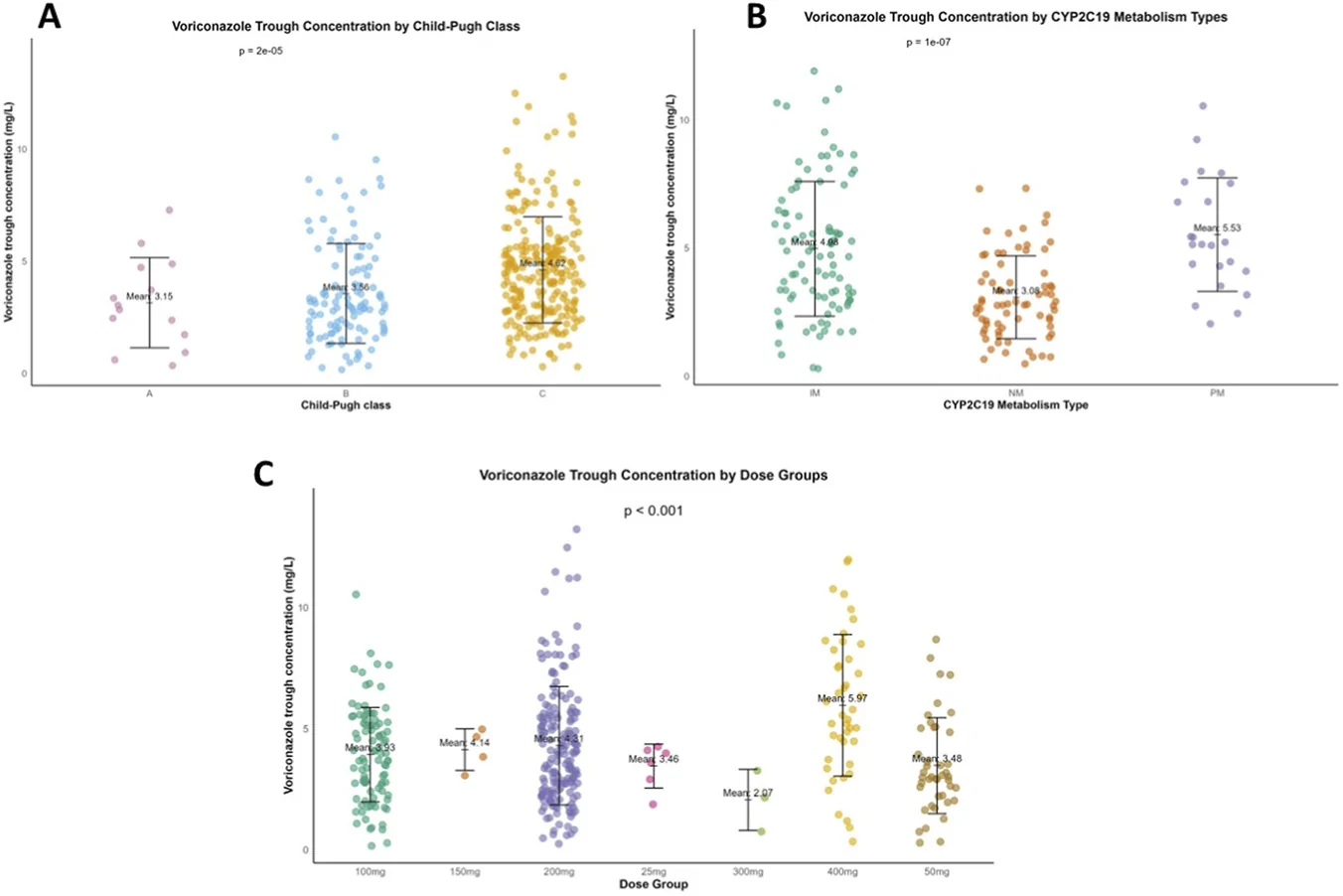
Voriconazole trough concentrations across different clinical and genetic subgroups. **(A)** Voriconazole trough concentrations stratified by Child-Pugh class. **(B)** Voriconazole trough concentrations stratified by CYP2C19 phenotype. IM, intermediate metabolizer; NM, normal metabolizer; PM, poor metabolizer. **(C)** Voriconazole trough concentrations stratified by daily dose group. Error bars represent standard deviations. P-values were calculated using the appropriate group comparison tests according to data distribution.

**FIGURE 3:**
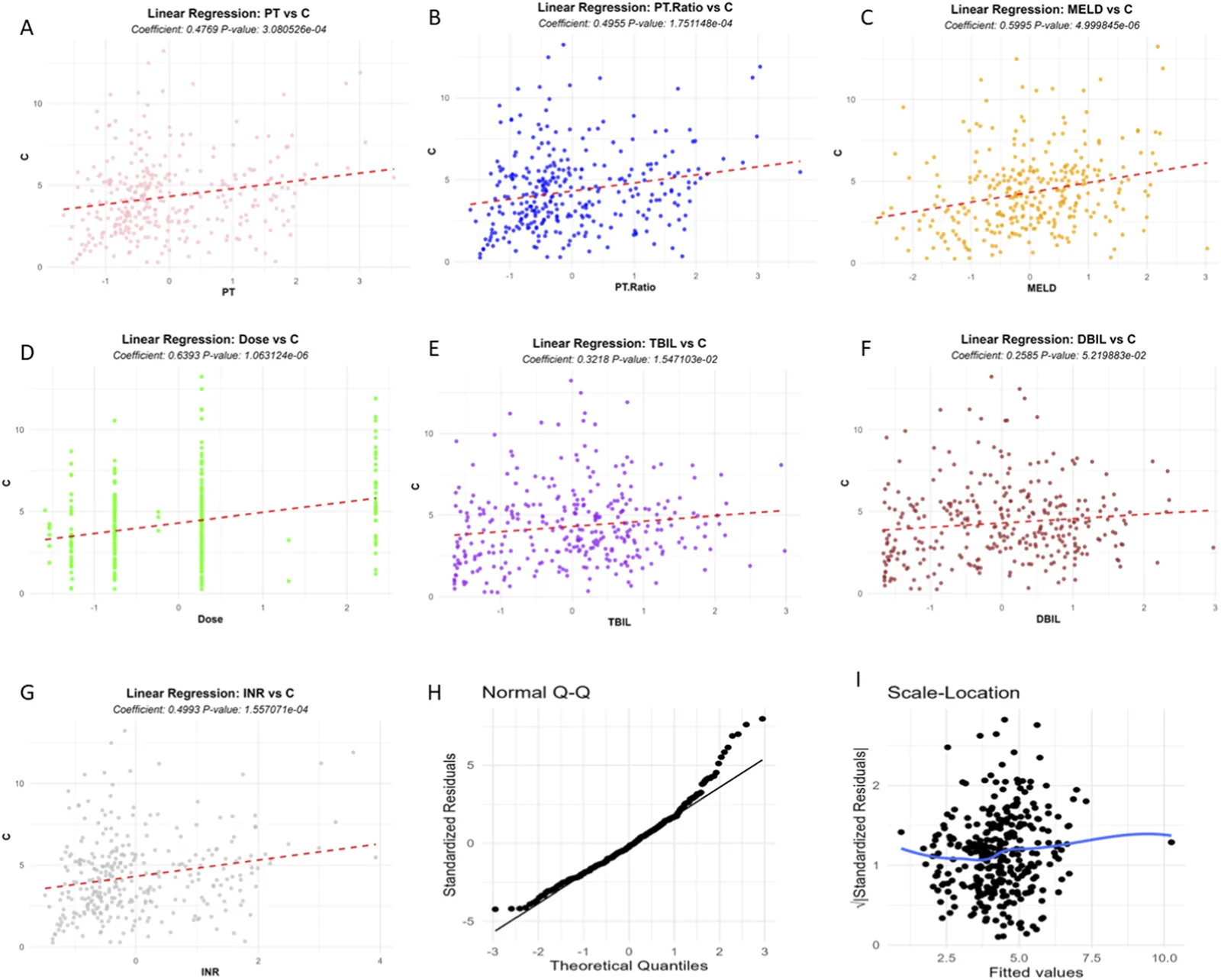
Linear regression analysis of factors associated with voriconazole trough concentrations. **(A)** Linear regression of prothrombin time (PT) against voriconazole trough concentrations. The coefficient was 0.4769 (P < 0.001), indicating a positive association. **(B)** Linear regression of PT ratio against voriconazole trough concentrations. The coefficient was 0.4955 (P < 0.001), suggesting a positive association. **(C)** Linear regression of MELD score against voriconazole trough concentrations. The coefficient was 0.5995 (P < 0.001), indicating a positive association. **(D)** Linear regression of daily dose against voriconazole trough concentrations. The coefficient was 0.6393 (P < 0.001), indicating that daily dose was associated with trough concentrations. **(E)** Linear regression of total bilirubin (TBIL) against voriconazole trough concentrations. The coefficient was 0.3218 (P = 0.015), indicating a positive association. **(F)** Linear regression of direct bilirubin (DBIL) against voriconazole trough concentrations. The coefficient was 0.2855 (P = 0.052), suggesting a borderline but not statistically significant positive association. **(G)** Linear regression of international normalized ratio (INR) against voriconazole trough concentrations. The coefficient was 0.4993 (P < 0.001), indicating a positive association. **(H)** Normal Q-Q plot showing the distribution of standardized residuals against theoretical quantiles. **(I)** Scale-location plot displaying standardized residuals *versus* fitted values.

The full cohort of 182 patients and 375 voriconazole trough concentration measurements was included in the analysis of factors influencing voriconazole exposure. Univariate correlation analysis showed that 13 covariates were significantly associated with voriconazole trough concentrations (Supplementary Table 2; Supplementary Figure 1). Differences in voriconazole trough concentrations across Child-Pugh classes, CYP2C19 phenotypes, and daily dose groups are shown in Figures 2A–C. Overall, higher voriconazole trough concentrations were observed in patients with more severe liver dysfunction and in CYP2C19 intermediate or poor metabolizers. In addition, voriconazole trough concentrations were associated with liver function, coagulation-related indicators, and routine blood parameters, as shown in Figures 3A–G.

#### 3.6.1 Multiple linear regression analysis including CYP2C19 phenotype

Thirteen covariates, including CYP2C19 phenotype, were entered as independent variables, with voriconazole trough concentration as the dependent variable. A multiple linear regression model was constructed using the stepwise method, and the results are shown in Table 4. In this model, 156 trough concentration measurements with complete CYP2C19 phenotype and covariate data were included. The adjusted R^2^ of the model was 0.290, indicating that the model explained 29.0% of the variation in voriconazole trough concentrations. The Durbin-Watson statistic was 1.424, and the model was statistically significant (F = 16.825, P < 0.001). The VIF values of all included covariates were less than 5, indicating no significant multicollinearity.

**TABLE 4:** Multiple linear regression analysis of voriconazole trough concentrations.

Variable	Unstandardized coefficients		Standardized coefficients	t	P-value	VIF
	B	Std. Error	β			
Model including CYP2C19 phenotype (n = 156)						
Intercept	3.744	0.486	​	7.695	<0.001	​
PTA	−0.040	0.009	−0.333	−4.663	<0.001	1.111
Daily dose	0.007	0.002	0.292	4.057	<0.001	1.129
CYP2C19 IM	1.679	0.359	0.344	4.673	<0.001	1.180
CYP2C19 PM	2.008	0.556	0.275	3.612	<0.001	1.263
R^2^			0.308			
Adjusted R^2^			0.290			
Durbin-Watson test			1.424			
F			16.825			
P-value			<0.001			
Model excluding CYP2C19 phenotype (n = 311)						
Intercept	2.079	0.783	​	2.654	0.008	​
Daily dose	0.009	0.001	0.382	7.143	<0.001	1.106
MELD score	0.071	0.020	0.244	3.539	<0.001	1.840
PTA	−0.023	0.009	−0.180	−2.602	0.010	1.840
R^2^			0.205			
Adjusted R^2^			0.197			
Durbin-Watson test			1.616			
F			26.417			
P-value			<0.001			

This table presents the regression analysis results for variables impacting trough concentrations with and without considering CYP2C19 polymorphism variations. The “Unstandardized Coefficients” represent the direct impact of each independent variable on the dependent variable, while the “Standardized Coefficients” express this impact in terms of standard deviations, facilitating comparison across variables. The “P-value” indicates the statistical significance of each coefficient, and the “VIF” (Variance Inflation Factor) assesses multicollinearity among predictors. The sample size in each model represents the number of trough concentration measurements included after excluding observations with missing covariate data. CI, confidence interval; OR, odds ratio.

The model formula is shown in Equation 1. With CYP2C19 normal metabolizers as the reference group, voriconazole trough concentration decreased by 0.040 mg/L for every 1% increase in prothrombin activity, increased by 0.007 mg/L for every 1 mg increase in daily dose, and increased by 1.679 mg/L and 2.008 mg/L in CYP2C19 intermediate and poor metabolizers, respectively.

$$\text{Voriconazole trough concentration }\left(\text{mg}/L\right)=3.744\;‐\;0.04\times \text{PTA }\left(\%\right)+0.007\times \text{Daily Dose }\left(\text{mg}\right)+1.679\times \text{CYP}2C19\text{ IM}+2.008\times \text{CYP}2C19\text{ PM}$$ (1)

CYP2C19 NM was used as the reference group; CYP2C19 IM and CYP2C19 PM were coded as dummy variables.

#### 3.6.2 Multiple linear regression analysis excluding CYP2C19 phenotype

After excluding CYP2C19 phenotype, the remaining 12 covariates were entered as independent variables, with voriconazole trough concentration as the dependent variable. A multiple linear regression model was constructed using the stepwise method, and the results are shown in Table 4. In this model, 311 trough concentration measurements with complete covariate data were included. The adjusted R^2^ of the model was 0.197, indicating that the model explained 19.7% of the variation in voriconazole trough concentrations. The Durbin-Watson statistic was 1.616, and the model was statistically significant (F = 26.417, P < 0.001). The VIF values of all included covariates were less than 5, indicating no significant multicollinearity.

The model formula is shown in Equation 2. Specifically, voriconazole trough concentration increased by 0.009 mg/L for every 1 mg increase in daily dose and by 0.071 mg/L for every 1-point increase in MELD score, but decreased by 0.023 mg/L for every 1% increase in prothrombin activity.

$$\text{Voriconazole trough concentration }\left(\text{mg}/L\right)=2.079+0.009\times \text{Daily Dose }\left(\text{mg}\right)+0.071\times \text{MELD Score }‐\;0.023\times \text{PTA }\left(\%\right)$$ (2)

### 3.7 Patient-level sensitivity analysis

To address the potential influence of repeated trough concentration measurements, a patient-level sensitivity analysis was performed using only the first available trough concentration from each patient. In the propensity score-matched cohort, the VALID group still showed a significantly higher initial target attainment rate than the control group [89.7% (35/39) vs. 48.7% (19/39), P < 0.001].

In the full cohort, patient-level regression analyses were further performed using the first available trough concentration from each patient. In the model including CYP2C19 phenotype, PTA, daily dose, and CYP2C19 IM remained significantly associated with initial voriconazole trough concentration, while CYP2C19 PM showed a positive but non-significant association. In the model excluding CYP2C19 phenotype, daily dose and MELD score remained significantly associated with initial voriconazole trough concentration, while PTA showed a negative but non-significant association. Overall, the directions of the key predictors were generally consistent with the concentration-level analyses (Supplementary Table 4).

## 4 Discussion

This study aimed to validate the efficacy and safety of a TBIL-based voriconazole dosing regimen (VALID regimen) compared with routine clinician-directed dosing in patients with liver dysfunction. Our findings demonstrated that the VALID group achieved a significantly higher target attainment rate of voriconazole trough concentration (89.7%) compared to the Control Group (48.7%) (P < 0.001). Additionally, the median initial voriconazole trough concentration was significantly lower in the VALID group (2.46 mg/L) compared to the Control Group (4.13 mg/L) (P = 0.0004). Consequently, these results suggest that the TBIL-based dosing regimen improved target trough concentration attainment and reduced excessive voriconazole exposure, which is consistent with previous studies emphasizing dose optimization in patients with liver dysfunction (Wu et al., 2024; Zhao et al., 2023; Lin et al., 2022; Chen et al., 2021). However, the difference in dose adjustment between the two groups was not statistically significant. Therefore, the benefit of the VALID regimen should be interpreted primarily as improved target attainment and safety, rather than as a significant reduction in the need for subsequent dose adjustment.

The safety profile of the TBIL-based regimen was also favorable. The incidence of voriconazole-related adverse events was significantly lower in the VALID group than in the control group [15.4% (6/39) vs. 38.5% (15/39), P = 0.022]. The most common adverse event was discontinuation due to excessive trough concentration, which was more frequently observed in the control group. These findings are consistent with previous studies showing that excessive voriconazole exposure is associated with an increased risk of adverse events and that careful dose optimization may improve treatment safety.

From a clinical perspective, the TBIL-based dosing strategy may provide a practical reference for initial voriconazole dose selection in patients with liver dysfunction. TBIL is routinely available in clinical practice and can be easily used for preliminary dose stratification (Chiang et al., 2024; Melenotte et al., 2023; Xiao et al., 2023). However, this strategy should not be considered a replacement for TDM. During voriconazole therapy, dose adjustment should still be individualized according to trough concentration, liver function changes, CYP2C19 phenotype when available, adverse events, and clinical response. The observed difference in initial maintenance dose distribution should be interpreted as the intended contrast between a TBIL-stratified initial dosing strategy and routine clinician-directed dosing practice. This finding supports the clinical rationale of the VALID regimen: reducing excessive early exposure while maintaining comparable clinical efficacy.

Besides, factors influencing voriconazole trough concentrations were further explored through correlation analysis and multiple linear regression analysis. In the multivariable model including CYP2C19 phenotype, PTA, daily dose, CYP2C19 IM, and CYP2C19 PM were retained as significant predictors of voriconazole trough concentration. In the model excluding CYP2C19 phenotype, daily dose, MELD score, and PTA were retained as significant predictors. These findings indicate that voriconazole exposure in patients with liver dysfunction is jointly influenced by dosing intensity, coagulation status, liver disease severity, and genetic metabolic capacity. In particular, the impact of CYP2C19 genetic polymorphisms on voriconazole metabolism was significant, with CYP2C19 IM and PM phenotypes associated with higher trough concentrations compared with CYP2C19 NM phenotype. These findings are consistent with previous studies highlighting the important role of CYP2C19 in voriconazole metabolism and support the consideration of pharmacogenetic factors in individualized dose adjustment when genotype or phenotype information is available (Zhao et al., 2021a; Li et al., 2023; de Jong et al., 2023; Zubiaur et al., 2023). In addition, the association of MELD score and PTA with voriconazole trough concentration further suggests that hepatic functional reserve and coagulation status may affect voriconazole exposure in patients with liver dysfunction. Therefore, TDM remains essential to prevent excessive exposure, reduce toxicity, and optimize dosing during treatment (Wang et al., 2022; Zhao et al., 2023; Yang et al., 2022).

Inflammation may also contribute to variability in voriconazole exposure, as inflammatory markers can affect drug metabolism and complicate dose adjustment in patients with liver dysfunction (Boglione-Kerrien et al., 2023; Liang et al., 2022; Li et al., 2022).

This study has several limitations. First, this was a single-center observational study with a relatively small sample size, which may limit the generalizability of the findings. Second, patients were not randomly assigned to the VALID group or the control group. Although propensity score matching was used to reduce baseline imbalance, residual confounding could not be completely excluded. In particular, the control group reflected routine clinician-directed dosing rather than a standardized prespecified regimen, and potential dosing heterogeneity or selection bias may have remained. Third, part of the data collection and outcome assessment relied on retrospective medical record review; therefore, underreporting or misclassification of adverse events could not be fully excluded, and efficacy assessment was not blinded to treatment group allocation. Fourth, voriconazole trough concentrations were generally measured 3–7 days after treatment initiation or dose adjustment, so early exposure during the first few days of therapy could not be accurately characterized. Fifth, some patients had repeated trough concentration measurements. To address this issue, we performed a patient-level sensitivity analysis using only the first available trough concentration from each patient. The directions of the key predictors were generally consistent with the concentration-measurement-level analyses, although future studies with larger sample sizes may apply longitudinal or mixed-effects models to better characterize repeated TDM measurements over time. Finally, CYP2C19 genotype or phenotype information was not available for all patients, and the target trough concentration range of 0.5–5.0 mg/L was based on the Chinese guideline, whereas other guidelines recommend slightly different ranges. These factors should be considered when interpreting the results.

In summary, the TBIL-based VALID regimen showed potential as a practical initial dosing strategy for patients with liver dysfunction, but larger multicenter prospective studies with longer follow-up, more complete pharmacogenetic information, and longitudinal modeling of repeated TDM measurements are needed to further validate its efficacy and safety.

## 5 Conclusion

In conclusion, the TBIL-based VALID regimen showed potential to improve target trough concentration attainment and reduce voriconazole-related adverse events in patients with liver dysfunction while maintaining comparable clinical efficacy. However, this was a preliminary single-center real-world validation study, and the findings should not be interpreted as definitive evidence for routine recommendation. Voriconazole exposure in this special population is influenced by multiple factors, including liver function, CYP2C19 phenotype, coagulation-related indicators, daily dose, and other clinical conditions. Therefore, TDM and individualized dose adjustment remain necessary during treatment, and the VALID regimen should be further validated in larger multicenter prospective studies.

## Data Availability

The raw data supporting the conclusions of this article will be made available by the authors, without undue reservation.
