# Supplementary material for: VALID regimen of voriconazole optimizes therapeutic target attainment and minimizes adverse events in patients with liver dysfunction
Source: Front Pharmacol. 2026 Aug 12;17:1895947. doi: 10.3389/fphar.2026.1895947 (PMC13506883; doi:10.3389/fphar.2026.1895947)
Supplement: Supplementary file 1 [file Table1.docx]

Supplementary Table 1. Baseline characteristics of included patients before propensity score matching

| Parameters^1^ | VALID Group (n=39) | Control group (n=143) | *P*-Value |
| --- | --- | --- | --- |
| Gender (Male, %) | 27 （69.2%） | 117（81.8%） | 0.0865 |
| Child-Pugh class（A：B：C） | 2：17：20 | 5：32：104 | 0.0225 |
| MELD Score | 15.6±7.8^*^  （17.0，9.1~20.9） | 21.2±7.7^*^  （21.6，16.2~25.2） | 0.0002 |
| Age (Year) | 57.9±11.7^*^  （56.0，49.0~69.0） | 52.3±12.8^*^  （53.0，45.0~61.0） | 0.0154 |
| Body Weight (kg) | 62.3±13.1^*^  （64.0，52.0~71.0） | 63.5±12.9^*^  （64.0，53.0~70.5） | 0.6408 |
| ALT（U/L） | 101.4±295.4  （27.7，17.6~61.0） | 63.8±52.9  （47.0，28.5~86.8） | 0.0101 |
| AST（U/L） | 121.1±311.6  （55.7，28.1~97.7） | 102.5±148.9  （75.1，50.0~114.8） | 0.0221 |
| TP（g/L） | 59.2±8.4^*^  （57.5，52.6~65.0） | 58.8±8.0^*^  （58.4，53.5~63.7） | 0.7932 |
| ALB（g/L） | 29.4±4.5  （29.6，24.9~33.0） | 32.1±4.7^*^  （32.1，29.7~34.9） | 0.0040 |
| GLO（g/L） | 29.7±7.9^*^  （28.5，24.8~33.8） | 26.5±7.7^*^  （25.8，20.6~31.1） | 0.0218 |
| TBIL（μmol/L） | 114.5±144.0  （48.2，22.5~138.5） | 271.5±157.3^*^  （262.7，151.7~370.8） | <0.0001 |
| DBIL（μmol/L） | 78.4±105.2  （25.5，14.6~97.1） | 176.3±109.8^*^  （262.7，151.7~370.8） | <0.0001 |
| TBA（μmol/L） | 110.3±121.3  （74.6，24.1~172.9） | 238.9±189.7  （173.7，107.0~349.6） | <0.0001 |
| BUN（mmol/L） | 9.26±7.23  （7.11，4.58~9.49） | 8.00±6.69  （5.83，4.17~9.32） | 0.1638 |
| CREA（μmol/L） | 90.0±57.4  （65.8，58.9~101.5） | 88.6±61.1  （69.6，55.0~101.6） | 0.9235 |
| UA（μmol/L） | 284.5±197.5  （215.0，153.8~399.9） | 216.8±138.8  （165.5，123.4~275.0） | 0.0282 |
| PT（s） | 20.6±5.9  （19.6，16.9~22.3） | 22.8±6.8  （20.5，17.8~27.9） | 0.0548 |
| INR | 1.80±0.70  （1.69，1.37~1.97） | 2.06±0.82  （1.77，1.45~2.56） | 0.0537 |
| PTA（%） | 50.2±16.8^*^  （46.0，40.0~63.0） | 44.7±18.6  （44.0，29.0~57.0） | 0.0512 |
| PT ratio | 1.57±0.45  （1.50，1.28~1.70） | 1.74±0.53  （1.57，1.34~2.08） | 0.0484 |
| WBC（×10^9^/L） | 5.82±3.65  （4.69，3.43~7.29） | 7.04±4.93  （5.62，3.80~9.21） | 0.1987 |
| RBC（×10^12^/L） | 2.86±0.70^*^  （2.78，2.41~3.19） | 2.83±0.69  （2.72，2.29~3.26） | 0.7210 |
| PLT（×10^9^/L） | 93.7±66.5  （70.0，40.0~130.0） | 82.9±54.3  （70.5，48.8~99.0） | 0.9121 |
| HGB（g/L） | 92.2±23.5^*^  （90.0，74.0~104.0） | 92.5±20.2  （89.5，77.0~107.0） | 0.8171 |
| NEUT#（×10^9^/L） | 4.40±3.42  （3.20，2.28~6.04） | 5.38±4.35  （4.09，2.47~6.79） | 0.1449 |
| NEUT%（%） | 71.2±12.0^*^  （69.3，64.6~80.0） | 72.2±12.3^*^  （73.4，62.3~81.1） | 0.6611 |
| LYM#（×10^9^/L） | 0.91±0.56  （0.85，0.50~1.14） | 0.97±0.75  （0.80，0.60~1.17） | 0.7601 |
| LYM%（%） | 18.6±10.1^*^  （17.7，11.3~24.1） | 16.8±9.7  （14.5，9.2~23.0） | 0.2695 |
| PCT（μg/L） | 1.071±1.532  （0.330，0.158~1.545） | 1.168±1.558  （0.700，0.388~1.290） | 0.0964 |
| CRP（mg/L） | 41.27±46.99  （20.78，10.42~62.24） | 24.14±28.63  （17.85，6.94~27.85） | 0.1296 |

^1^ Mean ± SD (Median, IQR).

^*^ Shows that the variable is normal distribution.
