# Supplementary material for: VALID regimen of voriconazole optimizes therapeutic target attainment and minimizes adverse events in patients with liver dysfunction
Source: Front Pharmacol. 2026 Aug 12;17:1895947. doi: 10.3389/fphar.2026.1895947 (PMC13506883; doi:10.3389/fphar.2026.1895947)
Supplement: Supplementary file 2 [file Table2.docx]

Supplementary Table 2. Correlation between VRZ trough concentration and covariates

| Covariates | Correlation | *P*-Value |
| --- | --- | --- |
| Gender | 0.0629 | 0.2246 |
| Child-Pugh class | 0.2416^****^ | <0.0001 |
| MELD Score | 0.2694^****^ | <0.0001 |
| CYP2C19 phenotype | 0.4100^****^ | <0.0001 |
| Age | 0.0417 | 0.4208 |
| Body weight | -0.0412 | 0.4448 |
| Daily dose | 0.1924^***^ | 0.0002 |
| ALT | 0.0311 | 0.5585 |
| AST | 0.0831 | 0.1168 |
| TP | -0.0188 | 0.7228 |
| ALB | 0.0556 | 0.2951 |
| GLO | -0.0574 | 0.2794 |
| TBIL | 0.1986^***^ | 0.0002 |
| DBIL | 0.1806^***^ | 0.0006 |
| TBA | 0.1122^*^ | 0.0346 |
| BUN | -0.0093 | 0.8630 |
| CREA | 0.0713 | 0.1870 |
| UA | -0.0693 | 0.1995 |
| PT | 0.2035^***^ | 0.0002 |
| INR | 0.2133^***^ | 0.0001 |
| PT ratio | 0.2155^***^ | 0.0001 |
| PTA | -0.2143^***^ | 0.0001 |
| WBC | 0.0437 | 0.4037 |
| RBC | -0.1049^*^ | 0.0446 |
| PLT | -0.1308^*^ | 0.0121 |
| HGB | -0.0441 | 0.4000 |
| NEUT# | 0.0598 | 0.2540 |
| NEUT% | 0.0866 | 0.0982 |
| LYM# | 0.0161 | 0.7586 |
| LYM% | -0.0538 | 0.3050 |
| PCT | 0.1036 | 0.2216 |
| CRP | -0.0395 | 0.6362 |

^*^P<0.05, ^**^P<0.01, ^***^P<0.001, ^****^P<0.0001
